# Daily and Weekly “High Doses” of Cholecalciferol for the Prevention and Treatment of Vitamin D Deficiency for Obese or Multi-Morbidity and Multi-Treatment Patients Requiring Multi-Drugs—A Narrative Review

**DOI:** 10.3390/nu16152541

**Published:** 2024-08-03

**Authors:** Pawel Pludowski, Ewa Marcinowska-Suchowierska, Galymzhan Togizbayev, Zhanna Belaya, William B. Grant, Stefan Pilz, Michael F. Holick

**Affiliations:** 1 Department of Clinical Biochemistry, The Children’s Memorial Health Institute, 04-730 Warsaw, Poland; 2 Department of Internal Medicine and Geriatric Cardiology, Medical Centre of Postgraduate Education, 01-813 Warsaw, Poland; emarcinowska1@gmail.com; 3 Department of Geriatrics and Gerontology, School of Public Health, Medical Centre of Postgraduate Education, 01-813 Warsaw, Poland; 4 Department of Rheumatology, Kazakh National Medical University, 050000 Almaty, Kazakhstan; g.togizbayev@gmail.com; 5 The National Medical Research Center for Endocrinology, 117036 Moscow, Russia; jannabelaya@gmail.com; 6 Sunlight, Nutrition and Health Research Center, San Francisco, CA 94109, USA; wbgrant@infionline.net; 7 Department of Internal Medicine, Division of Endocrinology and Diabetology, Medical University of Graz, 8036 Graz, Austria; stefan.pilz@chello.at; 8 Section Endocrinology, Diabetes, Nutrition and Weight Management, Department of Medicine, Boston University School of Medicine, Boston, MA 02118, USA; mfholick@bu.edu

**Keywords:** vitamin D in high doses, 7000 IU per day, 30,000 IU per week, 50,000 IU per week, effectiveness, safety, intermittent doses

## Abstract

Daily vitamin D supplementation using higher than normal dosing (up to the upper limit value) and intermittent (once or twice per week) dosing were studied in patients with increased risk of vitamin D deficiency. Using a PubMed database, a thorough search for published randomized controlled trials and other studies was conducted, and the results were analyzed. This review provides an overview of the use of 7000 IU daily, 30,000 IU per week or twice weekly, and 50,000 IU weekly of vitamin D for obtaining and maintaining 25(OH)D concentrations of at least 30 ng/mL in patients at high risk of vitamin D deficiency. The abovementioned dosages should be considered in adults with obesity, liver disease or malabsorption syndromes, or multi-diseased patients, mainly seniors requiring multi-drug treatment, including drugs affecting vitamin D metabolism. The simple schedules of 7000 IU/day, 30,000 IU/week or twice weekly, and 50,000 IU/week for use by patients with an increased risk of vitamin D deficiency were provided for consideration. Without monitoring of 25(OH)D, daily doses of 7000 IU or intermittent doses of 30,000 IU/week should be considered for a prolonged time as prophylactic or maintenance doses, mainly in obese patients, patients with liver disease and patients with malabsorption syndromes. For the treatment of possible vitamin D deficiency without assessment of 25(OH)D in these groups, intermittent doses of 30,000 IU twice weekly or 50,000 IU per week should be considered for a 6–8-week period only. The higher daily doses or the intermittent doses suggested above are effective, safe and responsive based on patient’s preferences.

## 1. Introduction

Vitamin D is a mediator in the regulation of skeletal, calcium and phosphate metabolism and has already been shown to play an important role in musculoskeletal health as well as in the prevention of nutritional rickets, osteomalacia and osteoporosis. The expression of vitamin D receptors (VDRs) in human cells suggests an even more general, extraskeletal impact of vitamin D on human health [1]. Vitamin D may be important for various organs and tissues due to the presence of VDRs in almost every tissue and cell. Consequently, vitamin D deficiency might have detrimental effects on these organs. In line with this, a decrease in the concentration of 25-hydroxyvitamin D (25(OH)D—the main marker of vitamin D supply)—has been associated with many chronic diseases. It has been shown that low concentrations of 25(OH)D (Table 1) are associated with the risk of developing cancer, malabsorption syndromes, osteoporosis and other diseases, as well as complications characterized by bone metabolism disorders, autoimmune diseases, allergies, endocrine diseases, and obesity and its complications [1,2].

The high incidence of vitamin D deficiency in the world requires actions to improve this situation. General screening for vitamin D deficiency is not recommended; however, 25(OH)D testing is suggested in certain risk groups susceptible to vitamin D deficiency to determine the optimal vitamin D dosage regimen and ensure sufficient vitamin D intake [1]. Assessment of 25(OH)D values is suggested in obese people and in patients chronically treated with drugs affecting vitamin D metabolism (e.g., anticonvulsants, glucocorticosteroids, antiretrovirus medications, etc.); in patients with malabsorption syndromes (e.g., cystic fibrosis, inflammatory bowel diseases, bariatric surgery and radiation enteritis), liver failure, chronic kidney disease, osteomalacia, chronic musculoskeletal pain, hyperparathyroidism and autoimmune diseases (e.g., multiple sclerosis and rheumatoid arthritis); in older people (>65 years), especially in those who have a history of falls or non-traumatic fractures (osteoporosis); in patients with granulomatous diseases (e.g., sarcoidosis and tuberculosis); in patients with a 24-hydroxylase deficiency; in people with chronic infections; and in people with dark skin pigmentation [1]. In many of these cases, higher doses of vitamin D are required to achieve the target 25(OH)D value. Recently, the Endocrine Society published new guidelines available for clinicians suggesting against routine vitamin D empiric supplementation for prevention of disease (except in pregnancy and pre-diabetes) and screening of 25(OH)D in adults aged 18–74 years [3]. The “empiric vitamin D”, according to “technical remarks”, “include daily intake of fortified foods, vitamin formulations that contain vitamin D, and/or daily intake of vitamin D supplement (pill or drops)” [3]. Both seniors 75 years of age and older as well as children and adolescents appeared to not be subject to these suggestions related to prevention of disease due to hypovitaminosis D. Previous Polish guidelines published in 2023 and Central and Eastern European statements recommend considering 25(OH)D measurement in patients at risk, and in cases where this is not possible, dosing recommendations for the general population should be followed, considering the possible co-occurrence of obesity [1,4].

It should be emphasized that synthesis of vitamin D in the skin (limited by the use of sun protection creams, age, religion, seasons of the year, etc.); ingestion from the intestine, adipose tissue storage and release of fat-soluble vitamin D; liver status and its disorders or diseases; kidney status and its disorders or diseases; medications influencing the anabolic or catabolic arm of the vitamin D endocrine pathway; multi-morbidity that needs multi-treatment with multi-drugs, etc., are known to affect vitamin D dosing schemes and proper choice of dosage. These factors always should be considered during a medical visit of a patient looking for help, despite the rules of the evidenced-based medicine. Another quote from the recently published Endocrine Society guidelines [3] is the following: “Based on the panel’s best estimates of treatment effects in adults aged 50 years and older, the panel judged that any desirable effects of intermittent, high-dose vitamin D (compared to lower-dose, daily vitamin D) are likely trivial, while the anticipated undesirable effects are likely to be small”.

Due to the prevalence of vitamin D deficiency around the world and the positive effects of greater vitamin D supplementation in high-risk groups, the aim of this study was to evaluate the available data on daily 7000 IU doses and 30,000 IU/week or twice weekly and 50,000 IU/week intermittent doses of vitamin D in high-risk groups.

This narrative review, “likely trivial” with obvious limitations, was aimed to evaluate the maintenance/prophylactic dosing of vitamin D in higher daily doses – for specific patient’s condition (obesity, malabsorption, etc.), or therapeutic dosing in the intermittent weekly doses – due to patient’s preferences, effectiveness, adherence, and safety.

## 2. Methods

A narrative review of the literature focused on high vitamin D dosing for groups at risk of vitamin D deficiency was performed using the electronic database PubMed.gov. The aim of the analysis was to provide practical suggestions, not strict recommendations, based on search results as well as our medical experience, for vitamin D dosing in obese patients as well as those with multi-disease and on multi-drug therapy with or without 25(OH)D monitoring, recognized as groups at high risk of vitamin D deficiency. Therefore, a review was performed on vitamin D deficiency in obese patients, patients with liver disease or malabsorption syndromes, and in seniors with polypharmacy to investigate the current prevention and treatment trends in these groups and to see if possible suggestions or even guidelines are available. Then, the final target was to review studies of dosage regimens of 7000 IU per day for prevention and 30,000 IU per week or twice weekly and 50,000 IU per week used for treatment of vitamin D deficiency in the above groups of patients. In the final stage of the search of the PubMed database, words such as “cholecalciferol 7000 IU daily”, “cholecalciferol 30,000 IU” and “cholecalciferol 50,000 IU”, together with additional words such as “daily” and “weekly”, were used to identify research on adult populations at high risk of vitamin D deficiency. Randomized controlled trials, clinical trials, meta-analyses and all studies focused on the doses specified above were selected for analysis.

## 3. Results

### 3.1. Groups at Significantly Increased Risk of Vitamin D Deficiency

#### 3.1.1. Obesity—Prevention and Treatment of Vitamin D Deficiency

According to the World Health Organization, the problem of excessive body weight affects over 2 billion people and contributes to approximately 2.8 million deaths annually [5,6]. For example, in Poland, overweight (i.e., a body mass index (BMI) in the range of 25.0–29.9 kg/m^2^) can be diagnosed in 60% of adult Poles, and in people with very high cardiovascular risk it can be diagnosed in 85% [5,6]. Obesity (BMI ≥ 30.0 kg/m^2^) is currently diagnosed in over 20% of adult Poles, and the National Health Fund estimates that in 2028 the percentage of adults suffering from obesity in Poland will be 30% [7]. This means that each family doctor in Poland, with 2500 patients in his or her practice, takes care of 1500 overweight people, including 500 obese patients, and this number will increase 1.5-fold by 2028 [7].

Obesity, leading to over 200 complications affecting virtually all organs and systems in the human body, is a significant cause of disability and increased mortality, making it a significant social and economic problem [8]. The complications related to obesity include vitamin D deficiency, which requires treatment. Epidemiological studies indicate that the probability of satisfactory vitamin D supplementation in a patient is inversely proportional to their BMI value, and, conversely, the higher the BMI value, the greater the probability of vitamin D deficiency [9]. Based on meta-analyses, it was estimated that obesity increases the risk of vitamin deficiency by approximately 1.5 times [10]. The potential causes of the increased risk of vitamin D deficiency among patients with overweight or obesity include (a) limited cutaneous cholecalciferol synthesis associated with lower exposure to sunlight (lower physical activity and social exclusion), (b) abnormal eating habits, (c) accumulation/sequestration in adipose tissue (“fat trap”) and (d) disruption of hepatic hydroxylation of cholecalciferol at position 25 [11].

Vitamin D deficiency in obese patients is a factor that increases the likelihood of metabolic complications of this disease [12]. Conversely, prospective studies have estimated that adequate vitamin D supply protects overweight patients from developing type 2 diabetes (odds ratio: 0.6) [13]. Pathophysiological mechanisms linking vitamin D deficiency with an increased risk of carbohydrate metabolism disorders include the regulatory influence of the active metabolite of cholecalciferol—calcitriol (1,25(OH)_2_D)—on the expression of pro-inflammatory genes (which leads to the silencing of metabolic inflammation causing insulin resistance) and genes controlling insulin secretion [14]. Vitamin D supplementation at a dose of 25,000 IU/week improved insulin sensitivity by 50% in obese patients [15]. Meta-analyses have also shown a beneficial effect of vitamin D supplementation (up to 60,000 IU/week) on fasting glucose levels and percentages of glycated hemoglobin in patients with pre-diabetes [16]. For the record, 60,000 IU/week is a higher dose than 7 days * 7000 IU/day (49,000 IU/week).

Regular vitamin D supplementation is an essential element of comprehensive care for obese patients. The guidelines suggest that to achieve and maintain a 25(OH)D concentration >30–50 ng/mL [1], the supplemented dose should be adjusted to the patient’s BMI value, and, importantly, the daily dose of cholecalciferol should be on average 2–3 times higher than the dose used in people with normal body weight, up to 10,000 IU/day without additional control [1,17]. It should be emphasized, however, that even when using high doses of vitamin D, it is generally difficult to achieve the recommended serum 25(OH)D concentration in overweight and obese patients [1,18]. Until recently, this problem was associated primarily with the sequestration of vitamin D in excessive adipose tissue; currently, impaired hydroxylation of cholecalciferol at position 25, associated with fatty liver disease, is increasingly being documented [19].

When determining individual dosages, factors such as age, body weight, dietary intake of vitamin D, comorbidities and medications taken should be considered [1]. Obesity should be an important consideration when determining doses. Recommendations regarding supplementation in people diagnosed with obesity indicate the need to double or triple the daily dose of vitamin D compared to dosages in people without the above weight problems [1]. Therefore, obese people over 75 years of age, usually suffering from several morbidities and often treated with many drugs, including those affecting the metabolism of vitamin D, should regularly take 7000–10,000 IU/day (up to 250 µg/day). In the case of persistent vitamin D deficiency, doses should be increased accordingly and adjusted to the degree of deficiency, if assayed, using therapeutic dosing, i.e., 30,000 IU twice weekly or 50,000 IU weekly.

#### 3.1.2. Digestive Tract Diseases—Prevention and Treatment of Vitamin D Deficiency

Digestive system diseases are some of the strongest risk factors for vitamin D deficiency. Chronic liver diseases and malabsorption disorders of various etiologies should be mentioned. The most common causes of vitamin D deficiency related to digestive tract diseases are presented in Table 2.

Under physiological conditions, 25(OH)D is mainly produced in the liver because of hydroxylation of cholecalciferol at position 25; therefore, the synthesis of this metabolite is impaired in patients with chronic liver diseases. Vitamin D deficiency is found in over 90% of patients with liver failure, both in the case of damage to the liver during cholestatic diseases, including primary sclerosing cholangitis, as well as in liver cirrhosis of post-inflammatory or alcohol-related etiology [19,20,21,22,23]. The major cause of vitamin D deficiency in liver disease is, however, malabsorption due to lack of bile and other secretions that help the absorption of vitamin D into the lymphatic system [2]. In recent years, the number of patients with severe liver damage due to metabolic-associated fatty liver disease (MAFLD), which also leads to reduced 25-hydroxylase activity and impaired 25(OH)D synthesis, has been increasing rapidly. Therefore, patients with liver dysfunction can benefit by increasing their vitamin D intake 2–3-fold (i.e., 7000–10,000 IU/day), and this strategy is an effective and safe solution [1].

The absorption of fat-soluble cholecalciferol in the intestine is estimated at approximately 70% in healthy people; however, in people with intestinal absorption disorders, e.g., after bariatric surgery, or in patients with celiac disease, short bowel syndrome, pancreatic insufficiency or biliary cirrhosis, the efficiency of cholecalciferol absorption is limited, deteriorates significantly and may drop below fifty to several percent, depending on the disease [24]. Therefore, in patients with digestive system diseases, doses starting from 7000 IU/day and 10,000 IU/day as part of a prevention strategy and higher doses, for example, 30,000 IU twice weekly or 50,000 IU per week, as part of the treatment of vitamin D deficiency should be considered [1]. However, patients exposed to treatment dosing should be monitored for their vitamin D status at least once every 2–3 months until a stable dose has been established and then less frequently, usually once or twice a year.

#### 3.1.3. Prevention and Treatment of Vitamin D Deficiency in Elderly People—The Problem of Multi-Morbidity and Multi-Drug Use

Taking into account that, as a result of the physiological aging process, there are changes in the pharmacokinetics of vitamin D (i.e., decreased absorption from the gastrointestinal tract and distribution in the body and an increased amount in adipose tissue) as well as the fact that, at least in Poland, multi-morbidity affects almost 70% of the youngest seniors and 90% of the oldest seniors, which results in an increased rate of multi-drug (>10 drugs) users, it is understandable that the senior population requires different procedures for the prevention and treatment of vitamin D deficiency to younger people.

In the (sub-)population of healthy seniors, the most common cause of vitamin D deficiency is insufficient supply in the diet and reduced effectiveness of skin synthesis, associated with the development of atrophic skin lesions and a gradual decrease in the concentration of the vitamin D precursor 7-dehydrocholecalciferol [25]. As a result of these changes, the efficiency of skin synthesis is four times lower in people over 70 than in young people with the same exposure to the sun. Additionally, an increase in the amount of adipose tissue, including overweight and obesity, and a decrease in lean body mass with age increases vitamin D deficiency [26].

Vitamin D deficiency occurs more often and is much more severe in older people with multi-morbidities and multi-drug use (geriatric patients). This is due to the overlapping states of impaired bioavailability and/or pharmacokinetics of vitamin D, which are consequences of individual diseases and treatment of the physiological changes associated with aging. Reduced absorption of exogenous vitamin D from the gastrointestinal tract occurs in patients with malabsorption syndromes (e.g., patients with inflammatory bowel diseases and celiac disease and patients who have undergone bariatric surgery). Chronic liver diseases, including fatty liver disease, lead to impaired vitamin D absorption, hepatic 25-hydroxylation and reduced 25(OH)D production, whereas chronic kidney disease impairs 1-α hydroxylation and calcitriol synthesis. The highest prevalence of vitamin D deficiency is observed in elderly patients with fragility fractures. It is usually associated with bone mineralization defects. This group of patients also require higher doses of vitamin D.

Multi-drug use and unfavorable drug interactions occur frequently in older people, and these should be remembered and taken into account when preventing vitamin D deficiency. The drugs used by these patients can have an unfavorable effect on the pharmacokinetics of vitamin D, leading to its systemic deficiency, and can be divided into drugs that reduce absorption of vitamin D from the gastrointestinal tract and drugs that affect its metabolism [27]. The first category includes lipase inhibitors, which are widely used in the treatment of obesity, much more often in younger people than in seniors. They increase the excretion of fats in the feces from 5% to 30%, including fat-soluble vitamin D, leading to systemic vitamin D deficiency. The second category includes drugs that affect the metabolism of vitamin D, including anticonvulsants, glucocorticosteroids, HIV medications and statins. Anticonvulsants (antiepileptic drugs), such as carbamazepine, phenobarbital and phenytoin, but also gabapentin (used not only in the treatment of epilepsy but also in the treatment of chronic pain, including neuropathic pain), lamotrigine (used in psychiatry as a mood stabilizer) and valproic acid (used in bipolar disorder) increase the activity of 24-hydroxylase—a key enzyme for vitamin D catabolism. This leads to increased elimination of all vitamin D metabolites and symptomatic osteomalacic achiness in the muscles and bones. Chronic glucocorticosteroids, regardless of the route of administration, promote the development of hypovitaminosis D and steroid-induced osteoporosis. Glucocorticoids have been documented to increase 24-hydroxylase expression and 24-hydroxylase mRNA expression. Studies also indicate direct inhibition of liver 25-hydroxylase by glucocorticosteroids. Moreover, these drugs directly block vitamin D receptors. Statins widely used in the treatment of lipid disorders (e.g., atorvastatin, lovastatin and simvastatin) are metabolized in the liver with the participation of the CYP3A4 enzyme, which is part of cytochrome P450. CYP3A4 also participates in vitamin D metabolism and promotes vitamin D metabolism disorders, including increased elimination. 

It should also be noted that vitamin D deficiency may reduce the effectiveness of some medications. Thus, in patients with osteoporosis treated with bisphosphonates or denosumab, in the presence of vitamin D deficiency, the increase in bone mass is much lower than in people who undergo treatment and are vitamin D sufficient [28]. Thus, it is widely recommended to first treat vitamin D deficiency and to only start with osteoporosis drugs when 25(OH)D concentrations are sufficient.

It should be noted that in the geriatric population, in which multi-morbidities and multi-drug use are common, to reach and then maintain appropriate vitamin D status requires the use of doses starting from 7000 IU/day.

However, a dose of 30,000 IU twice per week or 50,000 IU weekly as a treatment scheme for 6–8 weeks should be considered for those suffering from multi-morbidity and multi-drug use. 

Regarding safety issues, vitamin D toxicity was most often observed in people who took very high doses without a prescription and medical control of 50,000–100,000 IU per day of vitamin D for several months to several years, depending on age and body weight and other factors described above [29].

### 3.2. Cholecalciferol Supplementation at a Dose of 7000 IU Daily

In a double-blind study, 52 people aged 18 to 50 years with obesity (BMI > 30 kg/m^2^) and a 25(OH)D concentration < 20 ng/mL were randomly assigned to a group set to receive 26 weeks of treatment with 7000 IU of vitamin D administered daily or a placebo group [30]. Body composition and fat distribution [e.g., subcutaneous fat (SAT) and visceral fat (VAT)], insulin resistance (HOMA-IR), blood pressure, plasma lipids and circulating inflammatory markers were assessed. Half-year (26-week) supplementation with 7000 IU/day resulted in an increase in the average 25(OH)D concentration from 13.2 ng/mL (33 nmol/L) to 44 ng/mL (110 nmol/L, *p* < 0.0001) and a reduction in the average parathyroid hormone (PTH) concentration from 5.3 to 4.5 pmol/L (*p* < 0.01) in the intervention group using 7000 IU/day of cholecalciferol. Supplementation did not cause changes in the examined adipose tissue parameters (SAT and VAT) compared with the placebo. Supplementation with 7000 IU/day also had no significant effect on insulin resistance, blood pressure, plasma lipids or any of the several inflammatory markers tested, including high-sensitivity C-reactive protein (hsCRP). In their conclusions, the researchers stated that increasing the 25(OH)D concentration because of the supply of vitamin D at a dose of 7000 IU/day for 26 weeks had no effect on obesity complications in adults with low initial 25(OH)D concentrations apart from correcting vitamin D deficiency [30]. Interestingly, after analyzing body composition, including data determined by dual X-ray absorptiometry and bone turnover markers, a significant increase in bone mineral density (BMD, g/cm^2^) in the forearm by as much as 1.6 ± 0.7% (*p* = 0.03) was revealed, which is not usually reported after 26 weeks, and a positive effect of this dose of cholecalciferol (7000 IU/day) on bone turnover in people with obesity compared with placebo was observed [31].

Vitamin D deficiency is common in HIV-infected patients and is associated with an increased risk of disease severity and morbidity. HIV-infected people were invited to participate in a study determining the safety and effectiveness of using 7000 IU of vitamin D daily for 12 months to achieve and maintain increased serum 25(OH)D concentrations and improve immune status [32]. This was a double-blind study in women with perinatal HIV infection (PHIV) and behaviorally acquired HIV infection (BHIV) (5.0–24.9 years). Safety, serum 25(OH)D concentration and immune status were assessed at baseline and at 3, 6 and 12 months. After 3, 6 and 12 months of the study, 25(OH)D concentrations were higher in the case of supplementation with 7000 IU/day compared with the baseline value and higher than in the placebo group (*p* < 0.05). At baseline and then at 3, 6 and 12 months, the placebo group had 25(OH)D concentrations of 17.0 ± 9.2 ng/mL, 17.7 ± 9.0 ng/mL, 18.4 ± 10.4 ng/mL and 16.9 ± 9.3 ng/mL, respectively. The treatment group using 7000 IU/day exhibited a marked increase in 25(OH)D. At baseline and at 3, 6 and 12 months, the respective 25(OH)D concentration values were 18.2 ± 8.4 ng/mL, 32.5 ± 13.6 ng/mL, 29.2 ± 14.4 ng/mL and 28.4 ± 19.8 ng/mL. In the group supplemented with a dose of 7000 IU/day, the percentage of naive T helper cells (naive Th%) in the treatment group was higher (*p* < 0.01), as was the percentage of T helper cells (CD4%), which increased with supplementation in subjects taking highly active antiretroviral therapy (HAART)—a factor that strongly affects the vitamin D metabolism pathway. Additionally, viral RNA titers were reduced (*p* ≤ 0.05). The change in 25(OH)D concentration recorded during the study was a predictor of changes in viral RNA titers after 3 and 12 months and CD4% values after 3 months (*p* < 0.05). Daily administration of 7000 IU of vitamin D for 12 months was safe in HIV-infected people and effectively increased 25(OH)D concentrations [32]. Supplementation improved some clinically important markers of HIV resistance in patients taking HAART [32]. Moreover, a review of several studies on the potential protective role of vitamin D supplementation at a dose up to 14,000 IU per day in HIV-1 infection showed that the most effective dose in restoring adequate 25(OH)D concentration was 7000 IU per day [33]. Optimal 25(OH)D concentrations (>30 ng/mL) after supplementation with 7000 IU daily were found in 80% of patients, with higher concentrations observed after 12 months of treatment [33].

The effects of vitamin D supplementation on the cardiovascular system were determined by assessment of arterial stiffness and autonomic nervous system activity in an RCT including overweight or obese people in Brazil [34]. Patients aged 40–70 years with BMIs in the range of 25.0–39.9 kg/m^2^ and 25(OH)D concentrations < 30 ng/mL were exposed to 7000 IU/daily for 8 weeks. At baseline, the vitamin D group and the control group had 25(OH)D concentration values of 22.8 ± 4.9 ng/mL and 21.7 ± 4.5 ng/mL (*p* = 0.590), respectively. After 8 weeks of treatment, the group taking 7000 IU daily, but not the controls, exhibited a significant increase in 25(OH)D values (*p* < 0.001), showing concentrations close to 35.6 ng/mL. This coincided with a significant reduction in systolic blood pressure (SBP) from a baseline value of 123 ± 15 mmHg up to 119 ± 14 mmHg (*p* = 0.019) and a reduction in alkaline phosphatase (213 ± 55 mg/dL to 202 ± 55 mg/dL, *p* = 0.012). The vitamin D-treated group showed no change in augmentation pressure (AP) or the augmentation index (Alx) after 8 weeks: AP: 8 mmHg at baseline and 8 mmHg after 8 weeks; AIx: 26 vs. 25% at follow-up, respectively (*p* > 0.05). The controls, conversely, showed an increase in augmentation pressure (AP: 9 vs. 12 mmHg, *p* = 0.028) and the augmentation index (AIx: 26 vs. 35%, *p* = 0.020) [34]. Furthermore, after 8 weeks, the vitamin D-treated group showed a significant increase in the parasympathetic nervous system index (PNSi) (−0.64 ± 0.94 at baseline and −0.16 ± 1.10 at follow-up, *p* = 0.028) and in the mean intervals between each heartbeat (R-R) (from 866 ± 138 ms at baseline to 924 ± 161 ms after the study, *p* = 0.026). This RCT revealed that daily treatment of 7000 IU was safe and effective both for restoring proper vitamin D status in overweight or obese patients and for improving blood pressure and autonomic balance [34].

### 3.3. The Use of Intermittent Doses of 30,000 IU Once or Twice per Week and 50,000 IU Weekly

In a randomized, placebo-controlled trial, 30,000 IU per week was given for 24 weeks to prevent worsening of aromatase inhibitor-associated musculoskeletal symptoms (AIMSS) in women starting letrozole therapy for breast cancer [35]. Pain, 25(OH)D concentration, quality of life, fatigue, disability and hand-grip strength were assessed at baseline and at 12 and 24 weeks. The median age of the 160 subjects (80/arm) was 61. The median 25(OH)D concentration value in the placebo group was 25 ng/mL at baseline, 32 ng/mL at 12 weeks and 31 ng/mL at 24 weeks. In vitamin D-treated women, 25(OH)D levels, as expected, appeared markedly higher, with respective medians of 22 ng/mL at baseline, 53 ng/mL at 12 weeks and 57 ng/mL at the end of the study (24 weeks) [35]. No serious adverse events were noted in the treated groups. At week 24, a higher percentage (51%) of women from the placebo group experienced worse AIMSS events. In the vitamin D-treated group, the prevalence of AIMMS events was lower (37%); however, the difference between the placebo and vitamin D-treated groups was not significant (*p* = 0.069). When the brief pain inventories (BPIs) were assessed and compared between these groups, the difference became significant. In the vitamin D-treated group, 39% revealed pain vs. 56% in the placebo group (*p* = 0.024). The authors, using a categorical pain intensity scale (CPIS), concluded that discontinuation of letrozole, disability from joint pain or worsening of joint pain—all assessed as AIMMS events—did not change significantly after 24 weeks, but post hoc analysis using a different tool—the brief pain inventory (BPI)—suggested the potential benefit of 30,000 IU weekly in reducing AIMSS [35]. The dose of 30,000 IU per week of oral cholecalciferol appeared safe and effective in achieving proper vitamin D status [35].

In another RCT study aimed at evaluating safety and efficacy in obtaining proper vitamin D status, two protocols of cholecalciferol dosing administered to 69 patients with 25(OH)D concentrations < 20 ng/mL were studied [36]. The first protocol introduced a dose of 30,000 IU weekly for 10 weeks with a mean 25(OH)D baseline concentration of 14.1 ng/mL ± 4.0. In the second protocol, where the baseline 25(OH)D value was 14.9 ng/mL, 30,000 IU was given twice weekly for 5 weeks. After 10 weeks of 30,000 IU weekly, 79% of patients had a 25(OH)D concentration of 30 ng/mL. The second protocol appeared to be more effective. All subjects (100%) in the group exposed to 30,000 IU of vitamin D twice per week had a 25(OH)D concentration of at least 30 ng/mL. The mean increase in 25(OH)D concentration values was significantly higher in the group exposed to 30,000 IU twice weekly for 5 weeks. In this group, the 25(OH)D concentration increased by 46.6 ± 1.9 ng/mL. In the group that received 30,000 IU only once per week but for 10 weeks, the increase in 25(OH)D concentration was lower: 38.6 ± 1.8 ng/mL (*p* = 0.003). Both protocols appeared to be safe and effective; however, a larger increase in 25(OH)D concentrations was noted for 30,000 IU given twice weekly for 5 weeks. Therefore, this dose was recommended by the authors as proper for quickly obtaining proper concentrations of 25(OH)D [36]. Of note, this study was performed in patients with normal or slightly increased body weight (BMI = 26 ± 5 kg/m^2^). The effectiveness of using these doses for restoring proper vitamin D status in obese patients had not previously been studied.

Obesity affects a significant proportion of the global population and has been associated with vitamin D deficiency. Low serum 25(OH)D concentrations appear to be very common in obese people. Body weight-related problems were shown to increase the risk of several life-threatening diseases due to co-existing low-grade inflammation. An adequate 25(OH)D concentration of 30–50 ng/mL [1], showing good vitamin D supply, appeared as an immunoregulatory factor with markedly decreased adipogenic effects. In a double-blind placebo-controlled randomized clinical trial, 44 obese subjects with vitamin D deficiency, i.e., 25(OH)D < 20 ng/mL, were assigned for 12 weeks into a vitamin D or a placebo group [37]. The vitamin D group was treated with 50,000 IU weekly, and the placebo group was treated with edible paraffin weekly. Additionally, both groups were exposed to a weight-reduction diet. The study aimed to evaluate the dose of 50,000 IU of vitamin D given once per week as an intervention to decrease fat mass and low-grade inflammation in vitamin D-deficient obese patients. %Fat mass; body weight; parathyroid hormone (PTH); calcium; 25(OH)D concentration; and toll like receptor 4 (TLR-4), interleukin-1β (IL-1β) and monocyte chemoattractant protein 1 (MCP-1) levels were assayed at the start as well as after the intervention. After 3 months of 50,000 IU taken weekly, circulating concentrations of PTH (*p* < 0.001), TLR-4 (*p* < 0.05), IL-1β (*p* < 0.05) and MCP-1 (*p* < 0.05) were significantly decreased compared with the baseline values in the vitamin D group. These observations were accompanied by a marked increase in 25(OH)D concentrations (*p* < 0.001), as expected after 3 months of intervention (50,000 IU/week). At baseline, the 25(OH)D concentration in the treatment group was 11.5 ± 5.5 ng/mL, and after 3 months, surprisingly, it reached only 26.1 ± 7.2 ng/mL. In the placebo group, the baseline value was 10.0 ± 5.1 ng/mL, and after 12 weeks it had not changed (11.2 ± 5.8 ng/mL, *p* = NS). In both the placebo and vitamin D groups, significant decreases in % fat mass, body weight and BMI were also noted (*p* < 0.05); however, in the vitamin D-treated group, more marked decreases in body weight (7.0 kg vs. 4.8 kg, *p* < 0.05), % fat mass (4.6% vs. 3.3%) and serum MCP-1 levels (77.0 pg/mL vs. 19.1 pg/mL) were observed compared with the placebo. The observed decreases in body weight, % fat mass and MCP-1, together with the increased 25(OH)D concentrations, in the vitamin D group suggested the potential role of cholecalciferol in treating synergistically low-grade inflammation, obesity and vitamin D deficiency using 50,000 IU per week [37].

A similar study was performed in Jordan. The study evaluated the possible impact of cholecalciferol and/or calcium on body weight reduction and metabolic profile markers in 45 obese Jordanian females with vitamin D deficiency [38]. The study comprised four groups. The first group was treated with vitamin D at a dose of 50,000 IU weekly for 12 weeks. In this group, the baseline 25(OH)D was 11.1 ± 0.5 ng/mL. The next group was treated with calcium (1200 mg daily) together with vitamin D (50,000 IU/week), with a baseline value of 12.0 ± 1.2 ng/mL. The third group was exposed to calcium (1200 mg daily). In this group, the baseline value was 12.7 ± 0.8 ng/mL. The fourth group was a control group, with a baseline 25(OH)D concentration of 11.9 ± 0.8 ng/mL [38]. Additionally, all groups were exposed to a weight-reduction diet. After a 3-month study, Subih and colleagues noted a significant reduction in metabolic profile markers (*p* ≤ 0.05), including triglycerides (0.53 ± 0.21 mmol/L), cholesterol (0.56 ± 0.20 mmol/L) and PTH (27.6 ± 8.9 pg/mL), as well as body weight (10.5 kg), body fat percentage (2.4 ± 1.7%), BMI (4.6 ± 2.0 kg/m^2^) and waist circumference (11.4 ± 8.9 cm), compared with the group treated only with daily calcium and the controls. Furthermore, after 3 months, the increase in 25(OH)D concentrations appeared to be significant in all treated groups, with final values of 41.3 ± 2.3 ng/mL in the vitamin D only-treated group (*p* < 0.001), 45.6 ± 3.1 ng/mL in the group treated with calcium and vitamin D (*p* < 0.001) and 17.5 ± 0.8 ng/mL in the calcium only-treated group (*p* < 0.001), while in the placebo group the increase was not significant (1.8 ± 1.1 ng/mL, *p* = NS) and the final 25(OH)D concentration was 13.7 ± 1.1 ng/mL. The intervention with 50,000 IU per week together with calcium appeared to increase body weight reduction and improve biomarkers of metabolism as well as provide an interesting schedule with no adverse effects for the treatment of vitamin D deficiency in obese women from Jordan [38].

In a single-blinded randomized clinical trial, 100 Iranian patients awaiting bariatric surgery to reduce severe obesity were treated for 7 weeks using weekly doses of 50,000 IU of vitamin D. All patients were vitamin D deficient or insufficient (25(OH)D < 30 ng/mL) [39]. In the group treated with 50,000 IU per week for 7 weeks, 25(OH)D concentration values increased markedly from 15.2 ng/mL to 32.9 ng/mL (*p* < 0.01). In the group treated with a single loading dose of 300,000 IU, the increase was lower, from 25(OH)D concentration values of 13.2 ng/mL to 24.7 ng/mL at the end of the study. The authors concluded that the proposed treatment with 50,000 IU of vitamin D weekly for 7 weeks before bariatric surgery in patients with severe obesity should be considered and that it appeared to be safe [39]. Other methods used in this study were not recommended by the authors. No adverse effects were reported [39].

Table 3 provides a summary of the studies considered, showing data on the effectiveness of the use of 7000 IU daily, 30,000 IU weekly, 30,000 twice per week and 50,000 IU weekly in obtaining proper concentrations of 25(OH)D. Although the populations, durations of supplementation with vitamin D, ages, BMIs (if available) and outcomes differed between the studies, all showed positive increases in 25(OH)D concentration values. Furthermore, none of the studies reported adverse events related to the use of vitamin D dosing.

Even in children with obesity living in the United States, the dose of 50,000 IU of cholecalciferol appeared to be safe and effective in correcting vitamin D status [40]. In a study of children aged 12.3 years with obesity (BMI of 31.6 kg/m^2^), a single dose of 50,000 IU followed by doses of 6000 IU or 10,000 IU per day were given for 16 weeks. Specifically, in the first group, the 50,000 IU single dose was followed by 6000 IU/day for 16 weeks. After the 16 weeks, 67% of children achieved 25(OH)D concentration values ≥ 40 ng/mL, with a mean increase in 25(OH)D concentrations of 23.2 ± 14.2 ng/mL. In the second group, the 50,000 IU single dose plus 10,000 IU given daily achieved 25(OH)D concentration values ≥ 40 ng/mL in 73% of studied children. The mean increase in 25(OH)D values in that group was 31.3 ± 20.1 ng/mL. Interestingly, in the group of children with obesity who were exposed to 6000 IU daily for 16 weeks, only 50% achieved 25(OH)D values of ≥40 ng/mL, with a mean increase of 27.8 ± 18.9 ng/mL. Finally, a single dose of 50,000 IU and then doses of 8000 IU/day were tested. In this group, up to 78.6% of children reached a 25(OH)D concentration of ≥40 ng/mL, with a mean increase of 40.1 ± 22.9 ng/mL. No serious adverse effects related to vitamin D dosing were reported [40], and this study concluded that a 50,000 IU single dose plus 8000 IU per day of vitamin D is safe and effective in increasing 25(OH)D concentrations in children and adolescents with overweight or obesity values ≥ 40 ng/mL. It appeared that children and adolescents with obesity can safely increase their 25(OH)D concentration values to the 40–60 ng/mL range [2], which seems necessary for an anti-inflammatory effect on the proposed 16-week dosing regimen [40].

Finally, in a randomized controlled trial of zoledronic acid in reducing clinical fractures and mortality after hip fracture, patients, including ones with unknown 25(OH)D concentrations, received a loading dose of vitamin D (50,000 to 125,000 IU given orally or intramuscularly) 14 days before the first infusion of zoledronic acid (5 mg) [41]. The treatment was effective and safe, its effects including fracture risk reduction and possible restoration of proper vitamin D status. Thus, patients after fragility fracture may benefit from a single treatment dose even when their 25(OH)D values are not available. 

## 4. Discussion

Vitamin D deficiencies are common in both the general population and risk groups, such as patients with musculoskeletal diseases, systemic connective tissue diseases, endocrine and metabolic diseases, malabsorption syndromes, overweight and obesity, chronic kidney disease, cancer, reduced immunity, and even diseases of the central nervous system, as well as glucocorticosteroid users. Several peer-reviewed studies have demonstrated the benefits of vitamin D supplementation for various risk groups, often given at markedly higher doses compared with recommendations for the general population. Overall, ensuring adequate vitamin D intake is crucial to maintaining optimal health across a wide range of conditions [42,43,44].

Although vitamin D supplementation may be beneficial, it is important to exercise caution due to the potential risks associated with higher doses. Over-supplementation may lead to rare side effects like hypercalcemia and hypercalciuria. Concomitant use of vitamin D and calcium supplements may also increase the risk of kidney stones without proper hydration of the organism. The recommended upper limit for daily intake of cholecalciferol for the prevention of vitamin D deficiency, at least in Poland, was set at 4000 IU per day for adults of normal body weight and 10,000 IU per day for obese people [1,4].

Treatment of vitamin D deficiency in otherwise healthy patients (the general population) with vitamin D up to 7000 IU per day should be enough to maintain the 25(OH)D concentration in the range of 40 to 70 ng/mL throughout the year. In vitamin D-deficient patients suffering from serious diseases, the dosage regimen should be more stringent compared to healthy individuals and considered as an adjunct treatment, but it should never replace standard treatment.

Even though several of the mentioned risk groups (Table 2; Table 4) suffer from hypovitaminosis D, currently, no guidelines are available for clinicians regarding vitamin D dosing, except in overweight and obese people. The guidelines recommend considering 25(OH)D measurements in patients at risk, and in cases where this is not possible, dosing recommendations for the general population should be followed—of course, considering the possible co-occurrence of obesity [1]. Due to the frequent occurrence of vitamin D deficiencies and some possible effects of greater vitamin D supplementation in risk groups, the aim of this study was to document the validity of using a dose of 7000 IU per day or 30,000 IU or 50,000 IU as intermittent weekly doses in the mentioned risk groups, which should be considered absolutely safe. We propose that dosage regimens of 7000 IU/day or 30,000 IU/week or twice weekly or 50,000 IU/week be considered by medical doctors for the risk groups listed in Table 2; Table 4, even without a 25(OH)D test.

In clinical practice, healthcare professionals often consider prescribing vitamin D even without the result of a 25(OH)D test. In everyday life, patients have the right to medical treatment with appropriately high doses of vitamin D. Therefore, we have proposed a simplification due to the benefits of using different doses of vitamin D for the high-risk groups mentioned in Table 2; Table 3 and a higher dosing for consideration, starting from 7000 IU/day up to the 50 000 IU/week (equivalent of 7000 IU/day given for 7 days, approximately), especially for obese patients or for multi-morbidity and multi-treatment patients or patients with any combination of these medical conditions. Treatment with high-dose vitamin D formulations in matrix form (50,000 IU/week), due to the fast achievement of expected therapeutic effects, good tolerability, the safety of the drug and the convenience of intermittent administration to achieve greater patient compliance, should be considered in the abovementioned risk groups by medical doctors.

This study has some limitations. Firstly, the search for clinical studies and RCTs on the use of 7000 IU daily or 30,000 IU weekly or twice a week or 50,000 IU per week performed in groups at high risk of vitamin D deficiency, including obese patients or patients with liver disease or malabsorption syndrome or patients with multi-morbidity and multi-treatment requiring polypharmacy, revealed, very often, a limited number of studies or even a complete lack of results. For example, when we tried to investigate the effectiveness of use 7000 IU per day in obese patients only one study was available in PubMed, so the comparison between different studies using 7000 IU/day or different doses provided in one study of obese patients was not possible. That having been said, Polish guidelines [1] and Central and Eastern European statements [4] have at least provided the suggestion for obese patients of using doses 2–3 times higher (up to 10,000 IU per day, equal to the UL value) than those proposed for the general population (with normal weight), which was previously evidenced by Ekwaru and colleagues ten years ago [45].

Further, there was a limited number of studies on 7000 IU vs. 30,000 IU treatments and even a complete lack of studies on 30,000 IU vs. 50,000 IU treatments comparing the different dosages in the context of determining their effectiveness in obtaining concentrations of 25(OH)D at expected values (>30 ng/mL), especially in the high-risk groups we chose for this review. We realize that in some medical conditions a coinciding status of vitamin D deficiency has been reported, as it has for obesity, malabsorption, liver diseases and polypharmacy, despite the discussion of correlation or association. However, the role of professional medical care and doctors is to fight against deficiencies, of whatever sort, including vitamin D deficiency, with the use of safe and properly dosed treatments. In the case of high-risk groups, the doses we have proposed for consideration are higher than normal but still lower than the upper limit of 10,000 IU/d, even for 30,000 IU twice per week (7 days X 8600 IU, approximately), and they are safe, at least according to published data and our medical experience. Of course, we are aware that *CYP24A1* and *SLC34A1* mutations, hypercalciuria, hypercalcemia, nephrolithiasis, nephrocalcinosis and a history of other types of vitamin D hypersensitivity in an individual or family members should always be excluded before starting high-dose treatments such as those we have indicated for consideration by medical doctors. The basis of this suggested approach is the understanding that the potential benefits of vitamin D in proposed dosing in the treatment and/or prophylactic or maintenance during a specific health condition course significantly outweigh the risk of vitamin D intoxication and need for regular monitoring of 25(OH)D.

## 5. Conclusions

This study provides data on the safety and efficacy of dosages of 7000 IU per day or 30,000 IU/week or twice weekly or 50,000 IU per week for patients at high risk of vitamin D deficiency, including those with absorption disorders, liver diseases, obesity, multi-morbidity and multi-treatment requiring polypharmacy. Without monitoring of 25(OH)D and keeping safety in mind, despite the lack of adverse events reported, the treatment of possible vitamin D deficiency with intermittent doses of 30,000 IU twice weekly or 50,000 IU per week of cholecalciferol should be considered for 6–8 weeks of initial treatment, after which lower doses, i.e., 7000 IU/day or 30,000 IU/week, should be introduced and considered for a prolonged time as prophylactic or maintenance doses. With these clear and simple vitamin D dosage suggestions, we can contribute to the overall reduction in vitamin D deficiency rates and, consequently, improve public health outcomes.

## Figures and Tables

**Table 1:** Target threshold values for 25(OH)D concentrations.

25(OH)D Concentration	Vitamin D Status
<20.0 ng/mL (<50 nmol/L)	Deficiency—requiring treatment
20.0–29.9 ng/mL (50–75 nmol/L)	Suboptimal status—requiring prevention
30.0–50.0 ng/mL (75–125 nmol/L)	Optimal status—requiring prevention *
40.0–60.0 ng/mL (100–150 nmol/L)	Preferred range—requiring prevention **
60.0–100.0 ng/mL (150–250 nmol/L)	Increased status, an area of potential benefits and risks
>100 ng/mL (>250 nmol/L)	Potential risk of toxicity symptoms from vitamin D overdose *
>150 ng/mL (>375 nmol/L)	Serious risk of toxicity (with hypercalcuria, hypercalcemia and reduced PTH) **

* According to Polish Guidelines [1]. ** According to the older Endocrine Society Guidelines (USA) [2].

**Table 2:** The most common digestive tract diseases leading to vitamin D deficiency—indications for the use of high dosing of vitamin D.

Liver Failure During	Malabsorption Syndromes During
Primary cholangitis (formerly, primary cirrhosis biliary)	Celiac disease
Primary sclerosing cholangitis	Crohn’s disease
Alcoholic hepatitis	Cystic fibrosis
Autoimmune hepatitis and overlap syndromes	Pancreatic exocrine insufficiency
Infections with hepatotropic viruses	Bariatric procedures
	Whipple’s disease
	Short bowel syndrome

**Table 3:** Effectiveness of using 7000 IU per day, 30,000 IU per week, 30,000 IU twice weekly and 50,000 IU per week in adults for obtaining proper vitamin D status.

Study	Population	Age, Years	BMI, kg/m^2^	Dose and Duration	Baseline 25(OH)D, ng/mL	Follow-Up 25(OH)D, ng/mL	Outcomes	Adverse Effects
Wamberg et al. [30,31]	Obesity vs. placebo	18–50	>30	7000 IU/d for 26 weeks	13.2	44.0	PTH decreased; BMD increased; changes in SAT, VAT, hsCRP, blood pressure, plasma lipid ns	Not reported
Stallings et al. [32]	HIV-infected vs. placebo	5–25	N/A	7000 IU/d for 12 months	18.2	At 3 months: 32.5; at 6 months: 29.2; at 12 months: 28.4	Naive Th% increased, CD4% increased, viral RNA decreased	Not reported
Faria et al. [34]	Overweight and obesity vs. control	40–72	25.0–39.9	7000 IU/d for 8 weeks	22.8	At 8 weeks: 35.6	SBP decreased, ALP decreased, PNSi increased, R-R increased	Not reported
Khan et al. [35]	Breast cancer vs. placebo	Mean age: 61	N/A	30,000 IU weekly for 24 weeks	22.0	At 12 weeks: 53.0; at 24 weeks: 57.0	AIMMS trended lower, BPI lowered	Not reported
Takacs et al. [36]	Clinical study, no placebo	No data	26	30,000 IU weekly for 10 weeks;	14.1	At 10 weeks: 52.7	Restored proper vitamin D status	Not reported
				30,000 IU twice weekly for 5 weeks	14.9	At 5 weeks: 61.5	Restored proper vitamin D status	Not reported
Lotfi-Dizaji et al. [37]	Obesity vs. placebo	18–59	35.1	50,000 IU weekly for 12 weeks	11.5	At 12 weeks: 26.1	BMI decreased, %FM decreased, PTH decreased, MCP-1 decreased, IL-1ß decreased, TLR-4 decreased	Not reported
Subih et al. [38]	Obesity	18–48	37.5	50,000 IU weekly for 12 weeks	11.1	41.3	Restored proper vitamin D status	Not reported
Sayadi Shahraki et al. [39]	Obesity before bariatric surgery	36	43.4	50,000 IU weekly for 7 weeks	15.2	32.9	Restored proper vitamin D status	Not reported

Abbreviations: ns—not significant; PTH—parathyroid hormone; BMD—bone mineral density (g/cm^2^); SAT—subcutaneous adipose tissue; VAT—visceral adipose tissue; hsCRP—C-reactive protein; SBP—systolic blood pressure; ALP—alkaline phosphatase; PNSi—parasympathetic nervous system index; R-R—mean intervals between each heartbeat; AIMMS—aromatase inhibitor-associated musculoskeletal symptoms; BPI—brief pain inventory; %FM—percent of fat mass; MCP-1—monocyte chemoattractant protein 1; IL-1ß—interleukin 1ß; TLR-4—toll like receptor 4.

**Table 4:** Groups at very high risk of vitamin D deficiency in adults—indications for the use of high doses of 7000 IU of vitamin D per day or intermittent doses of 30,000 IU weekly or twice weekly or 50,000 IU per week.

Risk Groups	Diseases, Comorbid Conditions and Lifestyles
Multi-diseases and multi-drugs	All/selected diseases or conditions listed below, with treatments
Obesity disease	Obesity and overweight (and dyslipidemia)
Absorption disorders	Exocrine pancreatic insufficiency (old age, pancreatitis, type 2 diabetes, etc.), inflammatory bowel disease (Crohn’s disease and ulcerative colitis), cystic fibrosis, celiac disease, bariatric surgery
Liver and bile duct diseases	Liver failure, liver cirrhosis, cholestasis, fatty liver disease
Endocrine and metabolic diseases/disorders	Type 1 and 2 diabetes, metabolic syndrome, hypoparathyroidism and hyperparathyroidism, hypertension, polycystic ovary syndrome
Eating habits and age	Veganism and other types of vegetarianism, low-fat diet, senior age with multi-morbidities and polypharmacy
Diseases of the musculoskeletal system	Rickets, osteoporosis, osteopenia, bone pain, myalgia, myopathy, muscular dystrophy, recurrent low-energy bone fractures, repeated falls, deformities
